# Advances in genome editing for improved animal breeding: A review

**DOI:** 10.14202/vetworld.2017.1361-1366

**Published:** 2017-11-21

**Authors:** Shakil Ahmad Bhat, Abrar Ahad Malik, Syed Mudasir Ahmad, Riaz Ahmad Shah, Nazir Ahmad Ganai, Syed Shanaz Shafi, Nadeem Shabir

**Affiliations:** 1 Division of Animal Biotechnology, Faculty of Veterinary Sciences and Animal Husbandry, Sher-e-Kashmir University of Agricultural Sciences and Technology of Kashmir, Srinagar - 190 006, Jammu and Kashmir, India; 2 Division of Animal Genetics and Breeding, Faculty of Veterinary Sciences and Animal Husbandry, Sher-e-Kashmir University of Agricultural Sciences and Technology of Kashmir, Srinagar - 190 006, Jammu and Kashmir, India

**Keywords:** animal breeding, clustered regularly interspaced short palindromic repeats /Cas9, genome editing, transcription activator-like effector nuclease, zinc finger nucleases

## Abstract

Since centuries, the traits for production and disease resistance are being targeted while improving the genetic merit of domestic animals, using conventional breeding programs such as inbreeding, outbreeding, or introduction of marker-assisted selection. The arrival of new scientific concepts, such as cloning and genome engineering, has added a new and promising research dimension to the existing animal breeding programs. Development of genome editing technologies such as transcription activator-like effector nuclease, zinc finger nuclease, and clustered regularly interspaced short palindromic repeats systems begun a fresh era of genome editing, through which any change in the genome, including specific DNA sequence or indels, can be made with unprecedented precision and specificity. Furthermore, it offers an opportunity of intensification in the frequency of desirable alleles in an animal population through gene-edited individuals more rapidly than conventional breeding. The specific research is evolving swiftly with a focus on improvement of economically important animal species or their traits all of which form an important subject of this review. It also discusses the hurdles to commercialization of these techniques despite several patent applications owing to the ambiguous legal status of genome-editing methods on account of their disputed classification. Nonetheless, barring ethical concerns gene-editing entailing economically important genes offers a tremendous potential for breeding animals with desirable traits.

## Introduction

Varieties of breeding methods across different breeding programs have been used to improve genetic merit of domesticated animals. Majority of breeding programs have aimed at enhancing the genetic worth of animals using conventional selection methods primarily based on phenotype [1]. However, the efficiency of such method depends on identification of high merit individuals, selection intensity, generation time, and continued genetic diversity or its conversion into short- and long-term genetic gains [2]. The advent of techniques such as mapping, sequencing, and analyzing DNA information at genomic level adds a new dimension to animal breeding [3]. Advances in genotyping and sequencing have enhanced our ability to explore livestock genomes that further our comprehension of genomic selection. These innovations have arguably been significant in animal breeding from the time when the best linear unbiased prediction was developed in the 1940s [4]. Recent advances in gene editing, a powerful tool to manipulate genome, and bears applications in animal breeding programs aimed at accelerating genetic gain have generated enthusiasm among animal breeders [5]. Gene editing allows specific deletions, additions, or allele alteration at unambiguous locations in a genome [2]. Such alterations, if made in zygotes or germ line cells, can be permanent and heritable. Recently, genome editing in many livestock species has been reported [6] such as myostatin (*MSTN*) gene editing for “double muscling” in pigs, cattle, and sheep [7], polled gene introduction in dairy cattle [8], and edits to confer resistance to porcine reproductive and respiratory syndrome virus and African swine fever virus in pigs [7,9,10]. In livestock, the majorities of the traits of interest are quantitative and are likely to be affected by the additive effect of a number of causal variants. However, all the attempts at genome editing in livestock have targeted particular edits to address simple traits being controlled by a small number of causal variants with large effects [2]. Recently, through the promotion of alleles by genome editing (PAGE), it has been established that even editing of fewer causal variants can double the rate of both short- and long-term genetic gain contrary to selection [5].

This review aims to highlight the recent developments in genome editing technologies and their probable role in selective breeding programs to augment livestock production.

## The Promises of Gene Editing and Genomic Selection

The introduction of genomic selection has been welcomed with a lot of enthusiasm to the extent that some breeding companies are mulling to redesign their breeding programs. Using genomic selection, the breeding values for selection candidates at birth with potentially greater precision than classical pedigree index can be predicted [3]. Consequently, animals can be selected at an early age which is anticipated to double the rate of genetic gains per year in some cases [11]. Genomic information holds exciting prospects for animal breeding that could play a significant role in framing breeding plans to maximize long-term genetic gains [12]. Evidence indicate that the use of genomic information in individual breeding schemes could exploit more of the benefits of genomic information.

Whole genome sequencing of animals has completely changed we look at and interpret the genomic organization and evolution. The availability of whole genome sequences enables genome editing (site-specific mutagenesis), to obtain desired gene sequences. On the other hand, resequencing allows identification of a number of markers as well as the analysis of germplasm allele diversity based on allele mining approaches [13]. Moreover, resequencing also leads to the identification of haplotype blocks that are significantly correlated with quantitative trait variations [14]. Further, low-cost sequencing technologies allow us to frame genetic diversity in agreement to the requirements of contemporary agriculture including quality animal breeding. Gene editing will complement conventional breeding programs by editing loci having large effects on economic traits [2].

## Genome Editing

Different methods for faster genetic improvement in production animals have been used in a variety of selective breeding plans. Genetic engineering, particularly livestock genome editing entailing economically important traits, has recently been attempted [1]. Genome editing has a potential to enhance medium- and long-term genetic gains by increasing frequency of favorable alleles in a population [2]. Since the 1970s, the idea of scripting or editing genomes has been worked on, and several significant advances in these techniques have been made in the recent times [15,16]. The concept of genome editing is theoretically simple; base pairs at specific locations can be erased, altered, or added. These changes if made in germ cells are permanent and can be inherited [2].

### Animal breeding and genome editing tools

Genome editing has shown potential to revolutionize the precision and specificity of editing genomic targets [17]. These tools could be employed to enhance productivity, disease resistance, breeding efficiency, and for generation of novel animal models [7]. Lately, the development of designer nucleases (zinc finger nucleases [ZFNs], transcription activator-like effector nuclease [TALENs], and clustered regularly interspaced short palindromic repeats [CRISPR/Cas9]) has enabled extremely efficient and more facile genome editing in different animal species [18]. ZFNs share the use of the FokI nuclease and the need for dimers but use a different protein-DNA recognition mechanism [19]. TALEN comprises of a modular array of TAL recognition sequences joined to a FokI nuclease [20]. These are implanted in pairs, one for each strand, and work as dimers to generate double-stranded breaks in specific DNA sequences. It has variants such as the MegaTAL which uses a combination of TAL arrays with a nuclease (Mega-nuclease) having site-specific cleavage, thereby increasing the overall specificity of the combination [21]. MegaTAL as a technology is still in infancy, although presently being high cost and complex, they can be a turnkey solution in the future. For now, these are probably best left to those focusing on developing and refining the methodology. ZFNs are more cumbersome when it comes to usage, with no compensatory advantages, as compared to TALEN. CRISPR/Cas9 is a very recent method of making precise cuts in the genome. It is derived from bacteria and archaea wherein they constitute a part of their viral defense system [22]. CRISPR/Cas9 system consists of CRISPR which binds to a guide RNA and an associated endonuclease (Cas9). Its binding specificity is therefore dependent on the strength of RNA–DNA interaction. The foremost advantage of CRISPR/Cas9 over TALEN-based technologies (Table-1) is its rapidity of production and extremely low cost [23]. Over the past 5 years, these editing tools have been used to successfully mediate the generation of 300 gene-edited pigs, cattle, sheep, and goats [22]. Recent reviews describe the potential to use these tools in food animals for agricultural purposes [24-27] and include detailed descriptions of their mechanics and relative efficiencies. This is because the goals of genetic improvement programs do not change with new tools; breeders tend to employ whichever breeding method(s) most effectively achieves progress toward their breeding objective(s). It is likely that gene editing will synergistically complement traditional breeding programs, rather than replace or radically disrupt them.

**Table-1:** Comparison between ZFNs, TALENs, and CRRISR/Cas systems for genome editing.

Features of Gene editing tools	ZFNs	TALENs	CRISPR/Cas9
Target DNA recognition	Protein–DNA	Protein–DNA	RNA–DNA
Key components	ZFFok I fusion protein	TALEFok I fusion protein	Guide RNA and Cas9 protein
Function mode	ZF proteins recognize target DNA sequences R dimerization of Fok I nucleases induces DSBs of DNA RDSBs are repaired by NHEJ or HDR	TALE proteins recognize target DNA sequences R dimerization of Fok I nucleases induces DSBs of DNA R BSDs are repaired by NHEJ or HDR	Guide RNA recognizes target DNA sequence next to a NGG motifRCas9 induces DSBs of DNARDSBs are repaired by NHEJ or HDR
Advantages	Highly efficient and specific	Highly efficient and specific	Highly efficient, easy to be constructed, and capable of editing multiple sites simultaneously
Disadvantages	Largescale screening, timeconsuming, and expensive to be constructed	Tedious and time consuming to be constructed	PAM motif next to target sequence required

DSB=Doublestrand break, NHEJ=Nonhomologous end joining, HDR=Homologydirected repair, ZFNs=Zinc finger nucleases, TALENs=Transcription activatorlike effector nuclease, CRISPR/Cas9=Clustered regularly interspaced short palindromic repeats

### Methods for Creating Gene-edited Animals

To become an important driver of genetic change, gene editing methods must seamlessly integrate with conventional animal breeding programs which require reliability and precision to germ line-edit animals for selection. Edits can be introduced through gene editing of somatic cells followed by somatic cell nuclear transfer (SCNT) cloning or injection of the gene editing reagents into zygotic cytoplasm of the next generation [28].

### SCNT

SCNT has been a major technique for delivering nuclease-mediated genetic alterations in livestock [26]. The advantage of SCNT is that the gene-edited cell line can be genotyped and/or screened before transfer into the enucleated oocyte to ensure that the desired edits, and no off-target edits, have occurred [29].

A number of gene-edited animals have been produced through SCNT cloning technique (Table-2) [7,8,18,28,30-37]. The first successful attempt in cattle was made by Tan *et al*. [8] by editing the polled gene through TALEN. This breakthrough opened a new window in cattle genome editing. Subsequently, a gene responsible for double muscling was mutated [30] which resulted in the birth of double-muscled cattle. The other success story is *Staphylococcus aureus*-resistant cattle which was produced through SCNT by knocking in the human lysozyme in bovine fetal fibroblasts [31]. Other examples include editing of bovine lactoglobulin (BLG), human lactoferrin [32], and *MSTN* gene [33] in goats. Pig genomes were also edited and propagated through SCNT cloning technique with respect to MSTN [18,34], RELA (v-rel avian reticulo-endotheliosis) [35], and Von Willebrand factor (vWF) gene [36]. Despite the achievements made through SCNT-editing method, certain drawbacks associated with cloning such as early embryonic losses, postnatal death, and birth defects cannot be ignored [38].

**Table-2:** Genome editing of different livestock species through different methods.

Gene editing method	Species	Target	References
Somatic cell nuclear transfer	Cattle	Polled	[8]
		MSTN	[30]
		hLYZ	[31]
	Goat	BLG, hLF	[32]
		MSTN	[33]
	Pig	MSTN	[34] [18]
		RELA	[35]
		vWF	[36]
Zygote editing	Cattle	MSTN	[7]
		PRNP	[35]
		BLG	[28]
	Pig	RELA	[35]
	Sheep	BMPR/FecB	[37]

MSTN=Myostatin, hLYZ=Human lysozyme, BLG=Bovine lactoglobulin, RELA=vrel avian reticuloendotheliosis viral oncogene homolog A, vWF=Von Willebrand factor, PRPN=Prion protein, BMPR/FecB=Bone morphogenetic protein receptor/Booroola fecundity

### Direct editing of Zygotes

Direct editing of zygotes is advantageous owing to its direct application in the next generation; however, all embryos are not edited. On an average, fewer embryos are required to edit a particular gene using this approach as compared to SCNT considering the losses associated with cloning [26]. Various animal genomes have been edited through this method such as cattle, sheep, and pig (Table-2) [7,8,18,28,30-37].. A study demonstrated that injection of TALEN mRNA into the zygote can produce *MSTN* gene-edited cattle and sheep [7]. The innovation is critical to the cattle industry in a sense that specific gene-edited off-springs can be produced through ovum pickup, *in vitro* fertilization, and zygote microinjection (OPU-IVF-ZM). Similarly, PRNP (prion protein) [35] and BLG genes [28] were successfully edited in cattle by directly injecting TALEN reagents into the zygote. Two studies have focused on the RELA proto-oncogene gene in pigs which has been proposed to play a role in resistance against African swine fever [10,35]. Carlson *et al*. [35] successfully produced pig embryos only, whereas Lillico *et al*. [10] managed to obtain several viable piglets carrying the desirable mutations for RELA gene. Moreover, more recently BMPR/FecB gene was disrupted in sheep embryos by injecting the CRISPR/Cas9 reagents into the zygote [37].

## Gene Editing Intersects Conventional Breeding

**Figure-1:**
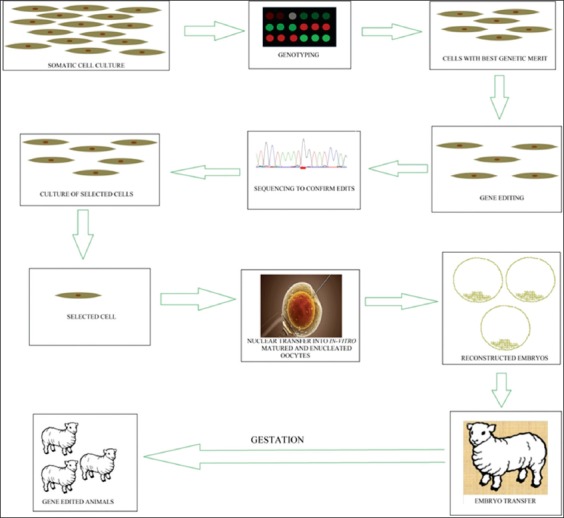
Integrating genome editing technology with reproductive technologies for producing high genetic merit progenies. Image modified from Van Eenennaam. (2017)

Worldwide, animal breeding organizations are heavily investing in genome editing technologies. Data generated out of some of the large-scale genome sequencing projects are revealing the association of nucleotide sequence changes with performance. It is anticipated that in the near future we can possibly directly edit genomes of low-producing animals for enhancing their genetic merit. Simultaneous targeting of different genes has permitted biallelic modification of up to three genes at the same time [39,40]. One could now possibly foresee editing of several alleles for different traits such as the known fertility impairing haplotypes [41], polled, and to correct known mendelian genetic defects that affect cattle [42], using conventional selection/breeding methods to keep making genetic progress in the direction of a given selection objective. Gene editing also offers a method to decode thousands of single-nucleotide polymorphism (SNP) markers which are revealed through sequencing projects and the discovery of causative SNPs into useful genetic variations for application in animal breeding programs [43]. One study has reported that conjoining gene editing with traditional genomic selection could lead to improvement in response to selection four-fold after 20 generations [5]. A potential approach to incorporate genome editing into current breeding programs through the use of advanced reproductive technologies is outlined in Figure-1.

Recent simulations have shown that genome editing for polygenic traits (PAGE) could double the genetic gains as compared to conventional genomic selection [5]. PAGE increases the frequency of favorable alleles although they segregate independently. Therefore, the favorable alleles can be fixed independently of the surrounding loci that would normally be coinherited. This coinheritance of favorable and unfavorable alleles leads to selection drag, resulting in delayed fixation of desired alleles or even their loss from the population. PAGE evades this drag by precisely targeting specific alleles and increasing the rate and the asymptote of genetic gain while as decreasing the level of inbreeding in genomic regions harboring favorable alleles. However, it is to be remembered that complex traits are characteristically impacted by many different genes. It is unlikely that all of the genes for such traits are known; also the desirable molecular edits for such genes are not typically evident. It is expected that genome editing in the future will be focused on the large effect loci and known targets to fix genetic defects or lower disease susceptibility, while as conventional selection will carry on to make progress in selecting for many small effect loci that impact the complex traits contributing to the breeding objective. In other words, editing will complement, not replace, conventional breeding programs.

## Regulatory Mechanism - A Policy Issue

Genome editing is not yet approved by governments, which is the first holdback to apply the technique widely in animal breeding. We are witnessing the timely convergence of technologies that together will have a significant influence on research, human health as well as in animal and plant breeding [44]. The exponential upsurge in the genome and expressed sequence data, the ability to compile, analyze, and mine these data through sophisticated bioinformatics procedures on high-powered processors, and progresses in various molecular and *in vitro* cellular techniques combine to shore up novel developments in commercial biotechnology and research. This promise can be fulfilled only if the regulatory oversight is impartial to the potential hazards and has an expansive support from consumers, researchers, and commercial interests [45]. Regulators and their advisors must appraise and apply novel ways of evaluating the future tidal wave of edited animals. Despite significant progress in gene editing, research, and development in most regions of the world, it still remains unclear as to how or whether this emerging technology will be regulated. The various risk management specialists and authorities as well as the biotechnology regulators have an exclusive opportunity to set up a rational, apt, and practical regulatory structure for gene editing that, unlike the GMOs, would ensure broad support from all the stakeholders.

## Conclusion

Genome editing is a new genetic engineering technology with a prospective future application for increasing the responses for quantitative traits in livestock breeding programs. The recently established engineered nucleases, namely, ZFNs, TALENs, and CRISPR/Cas9 enabled the precise modification of different animal genomes in a straightforward manner exemplifying the practical exploitation of genome editing technique. Genome-edited livestock is different from the traditional genetically modified animals as no recombinant DNA is incorporated into the animal genome, and hence, overcomes many of the questions associated with the production of genetically modified animals, thus increasing the prospect for its social acceptance. The integration of genome editing with industry standard and reproductive technologies will provide a practical approach for advancing livestock genetics and breeding.

## Authors’ Contributions

SAB and AAM: Drafted the manuscript, SMA: Reviewed the manuscript, RAS and NAG: Provided critical inputs, SSS: Assisted in drafting the manuscript, NS: Conceived the idea and reviewed the manuscript. All authors read and approved the final manuscript.
